# Metabolomics of Endurance Capacity in World Tour Professional Cyclists

**DOI:** 10.3389/fphys.2020.00578

**Published:** 2020-06-05

**Authors:** Iñigo San-Millán, Davide Stefanoni, Janel L. Martinez, Kirk C. Hansen, Angelo D’Alessandro, Travis Nemkov

**Affiliations:** ^1^Department of Human Physiology and Nutrition, University of Colorado Colorado Springs, Colorado Springs, CO, United States; ^2^Division of Endocrinology, Metabolism and Diabetes, Department of Medicine, University of Colorado Anschutz Medical Campus, Aurora, CO, United States; ^3^Department of Research and Development, UAE Team Emirates, Abu Dhabi, United Arab Emirates; ^4^Department of Biochemistry and Molecular Genetics, University of Colorado Anschutz Medical Campus, Aurora, CO, United States

**Keywords:** metabolomics, elite athletes, endurance, exercise, lactate, oxidative stress, amino acid metabolism, mitochondrial metabolism

## Abstract

The study of elite athletes provides a unique opportunity to define the upper limits of human physiology and performance. Across a variety of sports, these individuals have trained to optimize the physiological parameters of their bodies in order to compete on the world stage. To characterize endurance capacity, techniques such as heart rate monitoring, indirect calorimetry, and whole blood lactate measurement have provided insight into oxygen utilization, and substrate utilization and preference, as well as total metabolic capacity. However, while these techniques enable the measurement of individual, representative variables critical for sports performance, they lack the molecular resolution that is needed to understand which metabolic adaptations are necessary to influence these metrics. Recent advancements in mass spectrometry-based analytical approaches have enabled the measurement of hundreds to thousands of metabolites in a single analysis. Here we employed targeted and untargeted metabolomics approaches to investigate whole blood responses to exercise in elite World Tour (including Tour de France) professional cyclists before and after a graded maximal physiological test. As cyclists within this group demonstrated varying blood lactate accumulation as a function of power output, which is an indicator of performance, we compared metabolic profiles with respect to lactate production to identify adaptations associated with physiological performance. We report that numerous metabolic adaptations occur within this physically elite population (*n* = 21 males, 28.2 ± 4.7 years old) in association with the rate of lactate accumulation during cycling. Correlation of metabolite values with lactate accumulation has revealed metabolic adaptations that occur in conjunction with improved endurance capacity. In this population, cycling induced increases in tricarboxylic acid (TCA) cycle metabolites and Coenzyme A precursors. These responses occurred proportionally to lactate accumulation, suggesting a link between enhanced mitochondrial networks and the ability to sustain higher workloads. In association with lactate accumulation, altered levels of amino acids before and after exercise point to adaptations that confer unique substrate preference for energy production or to promote more rapid recovery. Cyclists with slower lactate accumulation also have higher levels of basal oxidative stress markers, suggesting long term physiological adaptations in these individuals that support their premier competitive status in worldwide competitions.

## Introduction

The physiological and metabolic response to exercise has fascinated scientists for centuries. In 1784, Antoine Lavoisier and Pierre-Simon Laplace designed an ice-calorimeter to measure the amount of heat emitted during combustion and respiration, which enabled the measurement of oxygen (O_2_) consumption during exercise (Underwood, 1944; Karamanou and Androutsos, 2013). Since then, maximal oxygen consumption (VO_2_max) has been considered the gold standard to measure cardiorespiratory fitness (Shephard, 1984). In the last two decades, the monitoring of physiological and metabolic response to exercise using this technique has become increasingly popular in the area of sports medicine and performance, fostered in part by studies involving elite professional athletes. These studies have shaped programs for individualized training, recovery and nutritional regimes and have been based traditionally on VO_2_max as the representative parameter due to its relatively easy measurement by indirect calorimetry using commercially available metabolic carts (Jensen et al., 2002).

Despite widespread use, laboratory physiological testing is not ubiquitous as not all athletes have access to advanced exercise laboratory facilities. In addition, respiration-based measurements are not always reproducible in monitoring performance, due to the multiple unmeasured variables that influence this the measured output. As such, additional markers of training status that are more acutely tied to cellular and tissue metabolism have been proposed. Lactate is one such metabolic biomarker that enables measurement of metabolic responses to exercise and serves as a surrogate for muscle stress. Although it had been considered a waste product of anaerobic metabolism, foundational studies by Brooks and colleagues began to shift this decades-old paradigm by demonstrating a higher degree of lactate consumption under fully aerobic conditions than previously thought using isotope tracing studies in rats (Brooks, 1986). Since then, lactate has been shown to be a major fuel source for the body, and even possesses hormone-like properties (Brooks, 2018).

Whole blood lactate measurement has been widely accepted as a valid method to evaluate the metabolic responses to exercise (Jacobs, 1986; Billat, 1996). This measurement also serves as a monitor of training status, since well-trained athletes have a higher lactate clearance capacity and decreased blood lactate levels (Donovan and Brooks, 1983; Bergman et al., 1999) likely due to enhanced mitochondrial function resulting from training (McDermott and Bonen, 1993; Dubouchaud et al., 2000). In addition to ease of measurement using portable equipment, these findings have promoted lactate monitoring as a viable readout for personalized athletic training programs.

To expand upon metabolic programs that may elicit lactate production, studies into substrate utilization (such as fat and carbohydrate oxidation) during exercise using indirect calorimetry have provided improved resolution of exercise physiology (Frayn, 1983). The subsequent combination of blood lactate measurement and substrate utilization during exercise has thus offered novel approaches to measure metabolic function and, indirectly, mitochondrial function and metabolic flexibility (San-Millán and Brooks, 2018). However, despite growing interest into the cellular responses to exercise, these techniques only enable the monitoring of a limited number of parameters thereby hampering our full understanding of metabolic responses in exercise physiology.

The field of metabolomics has emerged strongly in the last decade in many areas of scientific research as a powerful tool to precisely measure metabolic pathways at the cellular and systematic level (D’Alessandro, 2019). Chromatography-based separation of metabolites combined with improved scanning speeds and accuracy of mass detectors has greatly enhanced the breadth and depth of metabolite coverage monitored in a single experiment. These techniques are amenable to measuring metabolites within any biological matrix (Nemkov et al., 2017) and have been applied to multiple exercise-focused studies into metabolic response to exercise that varies by type, intensity, and duration (reviewed in Sakaguchi et al., 2019).

Given the predictive importance of lactate measurement in sports physiology and the robustness of mass spectrometry-based metabolomics, we hypothesized that identification of metabolic pathways altered in conjunction with lactate production will reveal molecular mechanisms of enhanced physical performance. To test this hypothesis, we measured metabolite levels in whole blood samples isolated from a team of elite professional cyclists before and after a graded maximal physiological test and compared metabolic profiles with respect to a lactate-dependent performance cutoff (PC). Expansion of knowledge around lactate-associated metabolic traits could serve to improve multiple aspects of individualized training, nutrition, overtraining, injury prevention and rehabilitation.

## Materials and Methods

Twenty-one international-level World Tour professional male cyclists (Tour de France Level) performed a graded exercise test to exhaustion on an electrically controlled resistance leg cycle ergometer (Elite, Suito, Italy). Physical parameters are included in Table 1. After a 15 min warm-up, participants started leg cycling at a low intensity of 2.0 W kg^–1^ of body weight. Exercise intensity was increased 0.5 W kg^–1^ every 10 min as previously described (San-Millán et al., 2009). Power output, heart rate and lactate were measured throughout the entire test and recorded every 10 min including at the end of the test. All study procedures were conducted in accordance with the Declaration of Helsinki and in accordance with a predefined protocol that was approved by all researchers and the Colorado Multiple Institutional Review Board (COMIRB 17-1281). Written informed consent was obtained from all subjects.

**TABLE 1:** Physiological parameters of male cyclists used in this study.

Age (year)	28.2 ± 4.7
Height (cm)	179.2 ± 7.6
Weight (kg)	70.7 ± 6.7
Body fat (%)	10.4 ± 0.7

### Blood Lactate Concentration Measurement

At the end of every intensity stage throughout the graded training period, a sample of capillary blood was collected to analyze both intra- and extra-cellular levels of L-lactate (Lactate Plus, Nova Biomedical, Waltham, MA, United States). Heart rate was monitored during the whole test with a heart monitor (Polar S725x, Polar Electro, Kempele, Finland).

### Subjects Group Classification – Performance Cutoff

Cyclists were separated into two groups based on the blood lactate concentration at an exercise intensity of 5.0 W kg^–1^, designated as the PC. Cyclists with PC lactate levels below the group average of 5 mmol L^–1^ were classified as the Gold group, while cyclists above the average were classified in the Silver group (Figure 2A).

### Metabolomics Assessment

#### Sample Collection

Whole blood samples were collected using the Touch-Activated Phlebotomy (TAP) device (Seventh Sense Biosystems, Medford, MA) as previously described (Catala et al., 2018). Due to a limited 100 μL sample volume collected with the TAP device, and to assess the utility of whole blood analyses with the goal of simplifying sample isolation by circumventing the need for centrifugation, whole blood samples were frozen in dry ice within 15 min of isolation and stored at -80°C until analysis. Prior to LC-MS analysis, samples were placed on ice and re-suspended with nine volumes of ice cold methanol:acetonitrile:water (5:3:2, v:v). Suspensions were vortexed continuously for 30 min at 4°C. Insoluble material was removed by centrifugation at 18,000 *g* for 10 min at 4°C and supernatants were isolated for metabolomics analysis by UHPLC-MS. The extract was then dried down under speed vacuum and re-suspended in an equal volume of 0.1% formic acid for analysis.

#### UHPLC-MS Analysis

Analyses were performed as previously published (Nemkov et al., 2017; Reisz et al., 2019). Briefly, the analytical platform employs a Vanquish UHPLC system (Thermo Fisher Scientific, San Jose, CA, United States) coupled online to a Q Exactive mass spectrometer (Thermo Fisher Scientific, San Jose, CA, United States). The (semi)polar extracts were resolved over a Kinetex C18 column, 2.1 mm× 150 mm, 1.7 μm particle size (Phenomenex, Torrance, CA, United States) equipped with a guard column (SecurityGuard^TM^ Ultracartridge – UHPLC C18 for 2.1 mm ID Columns – AJO-8782 – Phenomenex, Torrance, CA, United States) using an aqueous phase (A) of water and 0.1% formic acid and a mobile phase (B) of acetonitrile and 0.1% formic acid for positive ion polarity mode, and an aqueous phase (A) of water:acetonitrile (95:5) with 1 mM ammonium acetate and a mobile phase (B) of acetonitrile:water (95:5) with 1 mM ammonium acetate for negative ion polarity mode. The Q Exactive mass spectrometer (Thermo Fisher Scientific, San Jose, CA, United States) was operated independently in positive or negative ion mode, scanning in Full MS mode (2 μscans) from 60 to 900 *m/z* at 70,000 resolution, with 4 kV spray voltage, 45 sheath gas, 15 auxiliary gas. Calibration was performed prior to analysis using the Pierce^TM^ Positive and Negative Ion Calibration Solutions (Thermo Fisher Scientific). Acquired data was then converted from.raw to.mzXML file format using Mass Matrix (Cleveland, OH, United States). Samples were analyzed in randomized order with a technical mixture injected after every 15 samples to qualify instrument performance. Metabolite assignments, isotopologue distributions, and correction for expected natural abundances of deuterium, ^13^C, and ^15^N isotopes were performed using MAVEN (Princeton, NJ, United States) (Melamud et al., 2010). Discovery mode alignment, feature identification, and data filtering was performed using Compound Discoverer 2.0 (Thermo Fisher Scientific).

Graphs, heat maps and statistical analyses (either *T*-Test or ANOVA), metabolic pathway analysis, partial least squares discriminant analysis (PLS-DA) and hierarchical clustering was performed using the MetaboAnalyst 4.0 package (Chong et al., 2018). *XY* graphs were plotted through GraphPad Prism 8 (GraphPad Software Inc., La Jolla, CA, United States).

## Results

### The Whole Blood Metabolome Changes Significantly in Response to Exercise

**FIGURE 1:**
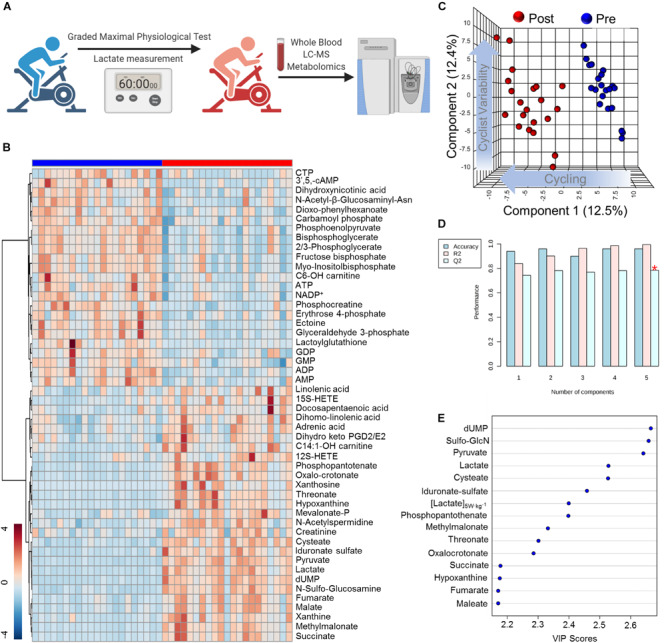
High-Throughput Metabolomics of Endurance Training in Elite Professional Cyclists. **(A)** Blood samples were drawn from subjects using a TAP^TM^ push-button blood collection device before and after 1 h of a graded exercise test. Samples were extracted for hydrophilic or non-polar metabolites and analyzed using a high-throughput LC-MS based metabolomics platform. **(B)** Hierarchical Clustering Analysis of the top 50 T-Test significant metabolites is shown as a heat map and are Z-score normalized, with the color-coded gradient depicted in the lower left corner. **(C)** Partial Least Squares Discriminant Analysis (PLS-DA) shows a distinct clustering pattern of baseline (Pre, blue) and post-test (Post, blue) samples along Component 1 axis (12.5% variance explained), while inter-individual variability is described by the Component 2 axis (12.4% variance explained). **(D)** PLS-DA cross validation details are shown for the first 5 components. **(E)** Parameter with the top 15 variable importance in projection (VIP) scores are shown.

In order to determine systemic metabolic changes during intense cycling, untargeted metabolomics analyses using LC-MS/MS were performed on whole blood samples isolated from cyclists before (Pre) and after (Post) a graded exercise test with cycling intensity normalized to individual cyclist body mass (Figure 1A). Of the 2,790 putative compounds detected in discovery mode analysis (ranging from Level 1 to Level 4 confidence, Schrimpe-Rutledge et al., 2016) 355 metabolites were manually identified using accurate intact mass, isotopic pattern, fragmentation and an in-house standard library. These metabolites were systematically analyzed by multivariate analyses such as Principal Component Analysis (PCA; Figure 1B) and Hierarchical Clustering Analysis (HCA, Figure 1C). A table including the molecular weight, retention time, polarity of detection, and raw peak area top values is provided in Supplementary Table 1. The top 50 significant metabolites by a two-tailed Student’s *T*-Test were hierarchically clustered to illustrate acute metabolic responses to cycling, showing significant changes for metabolites involved in central energy metabolism (glycolysis, TCA cycle), nucleotide homeostasis, and lipid metabolism (Figure 1B). Partially supervised PLS-DA distinguished samples by time point across Component 1 (12.5% of metabolic difference described) and demonstrated variability of metabolic responses amongst cyclists along the Component 2 axis (12.4% of the total metabolic difference described) (Figure 1C). The PLS-DA model demonstrated a high degree of accuracy using just the first component (*R*2 = 0.84, *Q*2 = 0.74, Accuracy = 0.92, Figure 1D). A larger degree of biological variability was noted in the Post time point cluster relative to the Pre time point, indicating that the cyclists’ responses to strenuous exercise varied within this group. The top 15 metabolites contributing to the clustering pattern include mitochondrial and glycolytic metabolites, indicating importance of these pathways (Figure 1E). A heatmap of metabolite values for the entire manually curated dataset is provided in Supplementary Figure 1.

### Metabolite Abundance Before and After Exercise Associates With Lactate Production in Response to Workload

**FIGURE 2:**
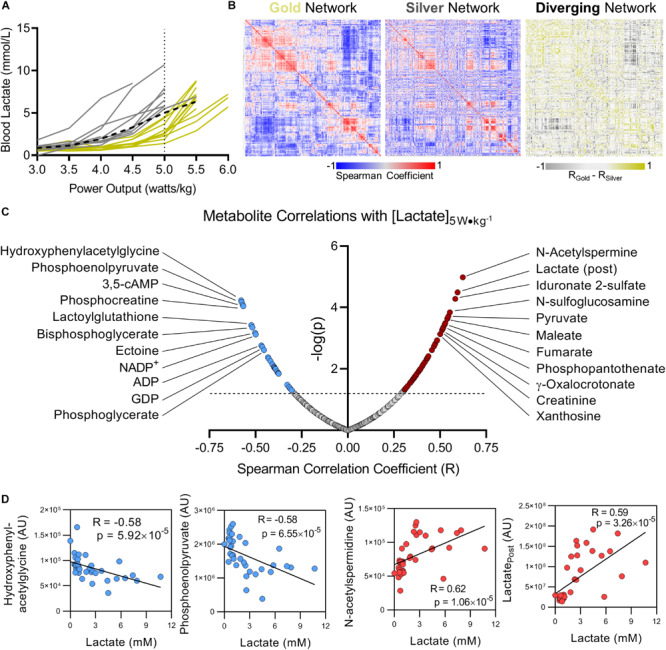
Blood lactate levels as a function of power output can metabolically distinguish cyclists. **(A)** Using blood lactate levels (in mM) at a power output of 5 W kg^–1^ (the maximum output of which most riders achieved, referred to as the performance cutoff, or PC), cyclists were assigned to the Gold or Silver performance groups for having blood lactate levels below or above the group average, respectively (group average indicated with a black dashed line). **(B)** A Spearman Rank Correlation matrix of metabolites measured in the Gold group was Hierarchically clustered and plotted as a heat map **(left)**. A Spearman Rank Correlation matrix of metabolites measured in the Silver group was then plotted according to the same hierarchical order determined for the Gold group **(center)**. The difference of Spearman correlation coefficients between the Gold and Silver group (Gold minus Silver) was then plotted as a heat map according to the same hierarchical order determined for the Gold group. Positive Spearman differences are colored gold, and negative Spearman differences are colored silver. **(C)** The top significant positive (red) and negative (blue) metabolite correlates with blood lactate concentration measured at 5 W kg^–1^ are shown, with the Spearman Correlation Coefficient (*R*) on the *x*-axis, and -log(*p*) on the *y*-axis. **(D)** Individual plots are shown for the top 2 metabolites with negative (blue, Hydroxyphenylacetylglycine and Phosphoenolpyruvate) and positive (red, N-acetylspermidine and Lactate post) Spearman correlations with blood lactate concentration measured at 5 W kg^−1^. Associated Spearman Correlation Coefficients and *p*-values are provided for each. Statistically significant correlations with log(*p*) > 1.3 (*p* < 0.05) are indicated above the dashed line.

Interval measurements of whole blood lactate levels in response to workload serves as a personalized metric for cycling performance (Jacobs, 1986). The training status of an individual cyclist can be determined in large part based on the power output (measured in watts per kilogram, W kg^–1^) at which lactate is produced more rapidly than it is consumed and begins to accumulate, also referred to as the lactate threshold (Brooks, 1985). As training status improves, the lactate threshold increases allowing the cyclist to exert a higher workload for longer periods of time prior to the onset of fatigue. Lactate levels in response to power output varied significantly amongst this group of cyclists. While all but 1 of the 21 cyclists monitored in this study were able to maintain a power output of 5 W kg^–1^, 14 cyclists reached 5.5 W kg^–1^, and only three cyclists were able to maintain 6 W kg^–1^ for 10 min (Figure 2A). To understand how the network between metabolic pathways differs in these cyclists (a concept we have previously referred to as metabolic linkage (D’Alessandro et al., 2017) we first classified the cyclists into two groups based on whether blood lactate concentration was higher (Gold) or lower (Silver) than the overall average at the designated PC of 5 W kg^–1^ (Figure 2A). Using the metabolomics dataset from the Gold group, a Spearman’s correlation matrix was prepared and hierarchically clustered to identify regions of the metabolome that are positively and negatively correlated (Figure 2B, left panel). A Spearman’s correlation matrix of metabolite levels in the Silver group was then prepared and plotted according to the same hierarchical order of the Gold group, and the difference in Spearman’s correlation coefficients for each relationship was calculated to highlight changes in metabolic networks between the two groups (Figure 2B, central and right panel, respectively). The Spearman’s correlation matrix values are provided in Supplementary Table 2. This metabolic network analysis revealed numerous significant associations between blood lactate concentration measured at 5 W kg^–1^ and metabolite levels before and after the fitness test (Figure 2C). Most of the significant correlations observed in this analysis are metabolites central to energy homeostasis and involved in glycolysis, the TCA cycle, purine homeostasis, and oxidative stress. While the top two negative correlates with lactate at 5 W kg^–1^ were the tyrosine catabolite 4-hydroxyphenylacetylglycine (*R* = -0.58, *p* = 5.92 × 10^–5^) and the late glycolysis intermediate phosphoenolpyruvate (*R* = -0.58, *p* = 6.55 × 10^–5^), the top two positive correlates with peri-test lactate production were the polyamine intermediate N-acetylspermidine (*R* = 0.62, *p* = 1.06 × 10^–5^) and post-test lactate (*R* = 0.59, *p* = 3.26 × 10^–5^) (Figure 2D). Furthermore, these two groups appeared to be distinguishable before the initiation of the graded-exercise test, suggesting that the metabolic status prior to exercise could theoretically predict performance (Supplementary Figure 2).

### Performance Cutoff Is Associated With Basal Oxidative Stress

**FIGURE 3:**
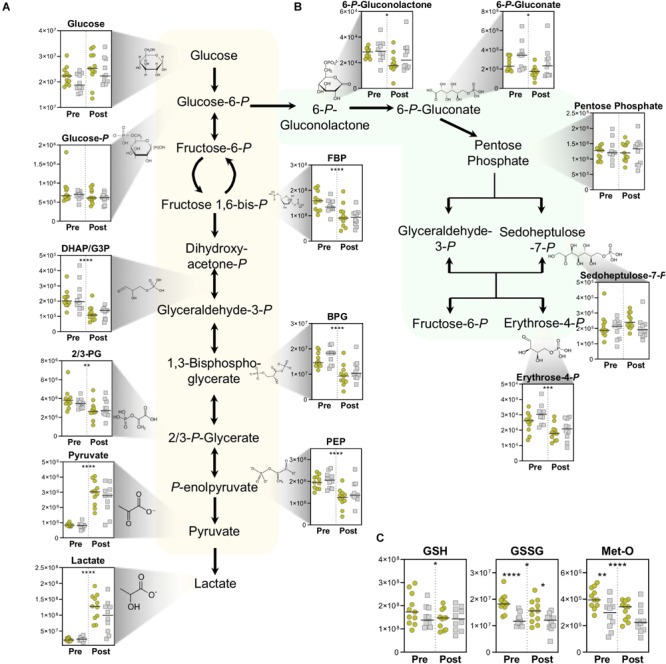
Energy and Redox Metabolism. Metabolite abundances (*y*-axis, Peak Area (AU)) are plotted for **(A)** Glycolysis, **(B)** pentose phosphate pathway, and **(C)** Reduced (GSH) and oxidized (GSSG) glutathione, as well as oxidative-stress derived methionine sulfoxide (Met-O). Samples from the Gold (°) and Silver (□) groups are shown Pre and Post exercise test (divided by a dotted line). *p*-values from a two-tailed paired *T*-test of comparisons between the Pre and Post time points using combined Gold/Silver group values are shown above the dashed line. p-values from a two-tailed unpaired homoscedastic *T*-test comparing the Gold and Silver groups at each time point are shown on the respective side of the dashed line. *p < 0.05; **p < 0.01; ***p < 0.001; ****p < 0.0001.

Lactate production is principally fueled by the metabolic routing of glucose through Embden–Meyerhof–Parnas glycolysis and the pentose phosphate pathway (PPP). In both the Gold and Silver groups, while whole blood glucose increased during exercise due to ongoing glycogenolysis for energy generation, all measured glycolytic intermediates significantly decreased in conjunction with accumulation of end-stage products pyruvate and lactate (Figure 3A). Although insignificant by *T*-test, glucose levels trend higher both before and after exercise, along with higher post-pyruvate and post-lactate levels in the Gold group, indicating an increased glycolytic capacity in these cyclists.

The PPP serves as a primary source of NADPH, which helps manage oxidative stress by functioning as a co-factor for glutathione reductase to convert oxidized glutathione (GSSG) to its reduced state reduced glutathione (GSH). Cycling resulted in overall decreases in PPP intermediates, indicating a preference of glucose metabolism through glycolysis for energy generation (Figure 3B). However, PPP intermediates 6-phosphogluconolactone, 6-phosphogluconate, and erythrose 4-phosphate were observed at higher levels overall in the Silver cycling group. This trend suggests increased activation of NADPH generating pathways to cope with the oxidative stress that arises during exercise. Interestingly, oxidative stress markers including oxidized glutathione (GSSG) and methionine sulfoxide (Met-O) were both significantly higher in the Gold group both pre- and post-test, suggesting alterations to oxidative stress and signaling pathways in these individuals (Figure 3C). Since handling of these samples was performed *in situ* in the exercise facility, a potential role of iatrogenic intervention on these measurements cannot be ruled out. As such, increased basal levels of these metabolites can be alternatively interpreted as a larger redox reservoir for the athletes in the Gold group at baseline and post-exercise.

### Lactate to Pyruvate Ratio Is Associated With Endurance Capacity

Through the activity of GADPH, NAD^+^ is reduced to NADH during the conversion of glyceraldehyde 3-phosphate into 1,3-bisphosphoglycerate. Under normal homeostasis in general, and especially in cases of high glycolytic flux that is required during high intensity exercise, lactate dehydrogenase oxidizes NADH back to NAD^+^ in the conversion of pyruvate to lactate, thereby maintaining necessary levels of the cofactor for the continuation of glycolysis. Cyclists in the Gold group had a higher post-test lactate-to-pyruvate ratio, which is proportional to NADH/NAD^+^ and a marker of glycolytic capacity. Interestingly, this ratio was lower at before the test, suggesting increased basal pyruvate oxidation capacity in Gold group cyclists due to larger mitochondrial networks (Figure 3A, lower right corner).

### Coenzyme A Synthesis Distinguishes Lactate Production Capacity

**FIGURE 4:**
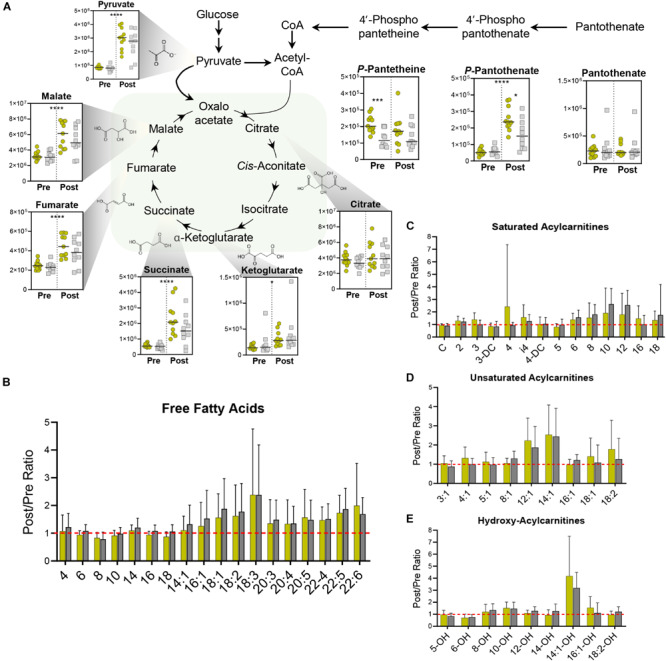
Mitochondrial Metabolism. **(A)** TCA Cycle metabolite abundances [*y*-axis, Peak Area (AU)] are plotted. Samples from the Gold (°) and Silver (□) groups are shown Pre and Post exercise test (divided by a dotted line). *p*-values from a two-tailed paired *T*-test of comparisons between the Pre and Post time points using combined Gold/Silver group values are shown above the dashed line. *p*-values from a two-tailed unpaired homoscedastic *T*-test comparing the Gold and Silver groups at each time point are shown on the respective side of the dashed line. Intra-subject ratios between the Post and Pre timepoints for both Gold and Silver groups are shown for **(B)** free fatty acids, **(C)** saturated acylcarnitines, **(D)** unsaturated acylcarnitines, and **(E)** hydroxy acylcarnitines. *p < 0.05; **p < 0.01; ***p < 0.001; ****p <0.0001.

In addition to its dehydrogenation into lactate for the regeneration of NAD^+^, pyruvate can also enter the mitochondria where it undergoes oxidative decarboxylation to form acetyl coenzyme A (acetyl-CoA). Exercise stimulated significant increases in many TCA cycle intermediates including α-ketoglutarate, succinate, fumarate, and malate (Figure 4A). The magnitude of increased TCA cycle intermediates in blood was larger in the Gold group, suggesting that these cyclists have a larger mitochondrial capacity to generate energy during exertion, possibly due to increased mitochondrial content in tissues such as skeletal muscle. In addition, this group demonstrated significantly higher levels of the coenzyme A (CoA) precursor 4’-phospho-pantetheine at baseline, and significantly higher levels of the upstream precursor, 4’-phospho-pantothenate after exercise. These trends point to enhancements in CoA synthesis as a metabolic adaptation that occurs in association with lower PC lactate levels.

In addition to its conjugation to carbohydrate-derived acetate, CoA also plays a critical role in mobilizing fatty acids for oxidation into acetyl-CoA. During aerobic periods of exercise, catecholamine and endocrine signaling results in the lipase-mediated liberation of fatty acids from di- and triacylglycerides stored in lipid droplets of muscle tissue and adipocytes. These fatty acids are chaperoned through circulation by albumin and transferred to tissues, where they are reversibly conjugated to carnitine and CoA for oxidation in the mitochondria. During the graded exercise test, all cyclists mobilized similar levels of free fatty acids (Figure 4B) and produced similar amounts of acylcarnitines (Figures 4C–E).

### Strenuous Exercise Depletes Energy-Rich Nucleoside Phosphates and Leads to Accumulation of Purine Degradation Products

**FIGURE 5:**
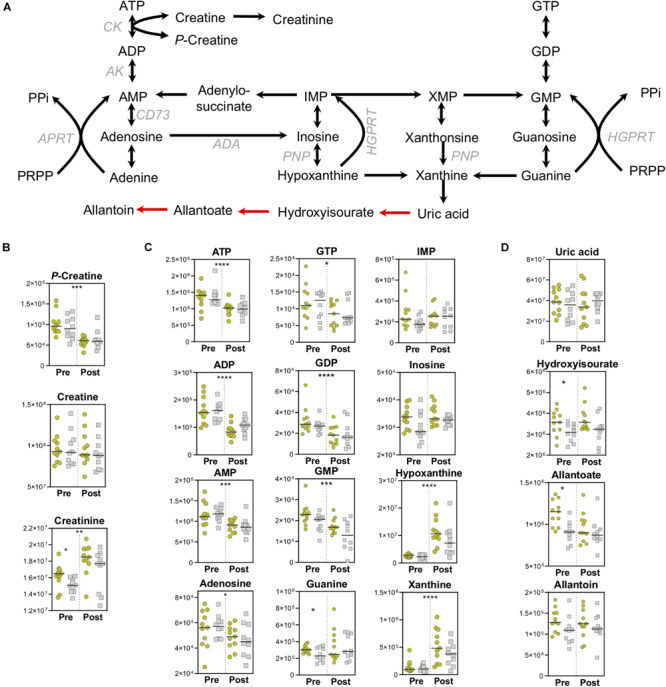
High-Energy Phosphates and Purine Metabolism. **(A)** A pathway map for high-energy phosphate and purine homeostasis is shown. Spontaneous, non-enzymatic reactions are shown in red arrows. Metabolite abundances [*y*-axis, Peak Area (AU)] are plotted for **(B)** Creatine homeostasis, **(C)** Purine salvage, and **(D)** Purine catabolism. Samples from the Gold (°) and Silver (□) groups are shown Pre and Post exercise test (divided by a dotted line). *p*-values from a two-tailed paired *T*-test of comparisons between the Pre and Post time points using combined Gold/Silver group values are shown above the dashed line. *p*-values from a two-tailed unpaired homoscedastic *T*-test comparing the Gold and Silver groups at each time point are shown on the respective side of the dashed line. *p < 0.05; **p < 0.01; ***p < 0.001; ****p < 0.0001.

As expected, high energy phosphate compounds are depleted during exercise. Utilization of phosphocreatine pools maintains a steady state of creatine, and fuels the significantly increased pools of creatinine (Figure 5B). The conversion of creatine to creatinine is an irreversible, non-enzymatic process that is favored in low-pH and high-temperature environments (Lempert, 1959) both of which onset during exercise. As creatinine levels serve as a rough measure of muscle mass (Virgili et al., 1994) higher basal levels in the Gold group indicate increased muscle mass that is associated with lower PC lactate (Figure 5B). In addition to dietary sources, creatine can also be synthesized from arginine by Arginine:Glycine amidinotransferase (AGAT), producing ornithine in the process. While arginine levels decrease overall during exercise, ornithine levels are higher at baseline in the Gold group, suggesting alterations to nitrogen metabolism that may serve to differentially fuel creatine pools (Supplementary Figure 2).

In like fashion to the depletion of phosphocreatine, high-energy pools of ATP and GTP are also utilized, resulting in increased levels of purine catabolites and intermediates of the salvage and deamination pathways, including hypoxanthine (Figure 5C). Despite increased levels of the purine catabolite xanthine, the end stage metabolite of enzymatic purine catabolism, uric acid, did not increase (Figure 5D). Although humans do not contain uricase, which converts uric acid into 5-hydroxyisourate and allantoin, this process can proceed spontaneously through the mediation of reactive oxygen species (ROS). As such, ROS-driven production of allantoin is a marker of oxidative stress in red blood cells (Kand’ár and Záková, 2008). Higher baseline levels of 5-hydroxyisourate and allantoate were observed in the Gold cyclists, suggesting higher levels of oxidative stress in these individuals that could be due to adapted metabolic pathways that result in lower PC lactate levels (Figure 5D).

### Amino Acid Utilization, in Addition to Performance Cutoff Can Distinguish Cyclists

**FIGURE 6:**
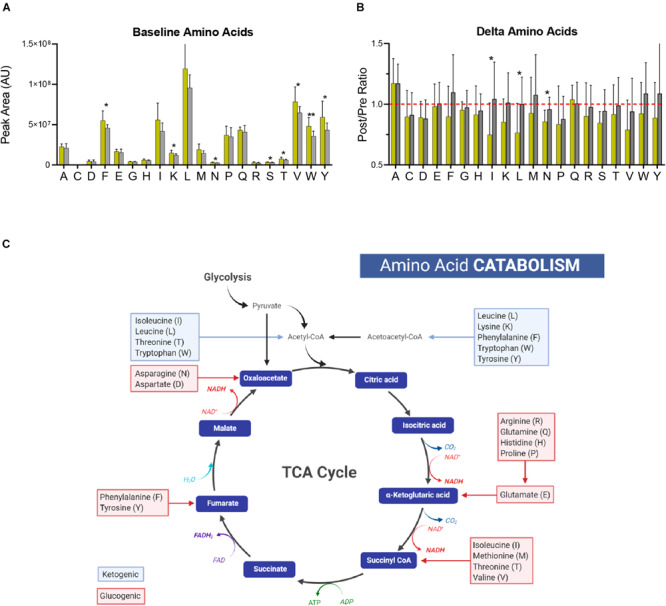
Amino Acid Catabolism. **(A)** The baseline (pre-training) levels of amino acids (labeled with single letter code) are shown for the Gold and Silver performance groups. **(B)** Fold changes of amino acid levels (Post/Pre) for both performance groups are shown. A ratio of 1 is indicated by a dashed red line. **(C)** A generalized schematic of amino acid catabolism through the TCA cycle is shown. Glucogenic amino acids, which have the capacity to fuel gluconeogenesis, are listed in red boxes. Ketogenic amino acids, which contribute to acetoacetate and acetyl-CoA pools, are listed in blue boxes. *p*-values from a two-tailed Student’s *T*-test are shown as *p < 0.05; **p < 0.01; ***p < 0.001; ****p < 0.0001.

In addition to energy and redox metabolites involved in glycolysis, the PPP, and the TCA cycle, amino acid levels at both baseline and after exercise differ based on PC lactate. At baseline, circulating levels of phenylalanine, lysine, asparagine, serine, threonine, valine, tryptophan, and tyrosine were significantly higher in Gold group cyclists (Figure 6A). In addition, the post-exercise levels of most amino acids were lower only in the Gold group, especially for isoleucine, leucine, and asparagine (Figure 6B). Given that elevated protein synthesis during this exercise period is unlikely to explain these changes due to both time- and energetic-constraints, it is possible that these amino acids are catabolized preferentially in the Gold group for ATP production (Figure 6C). In support, acylcarnitines involved in amino acid catabolism including C3-carnitine and C4-carnitine appear to increase more in this group (Figure 4C).

### Untargeted Metabolomics Identifies Distinct Tyrosine Metabolic Profiles in Relation to Performance Cutoff

**FIGURE 7:**
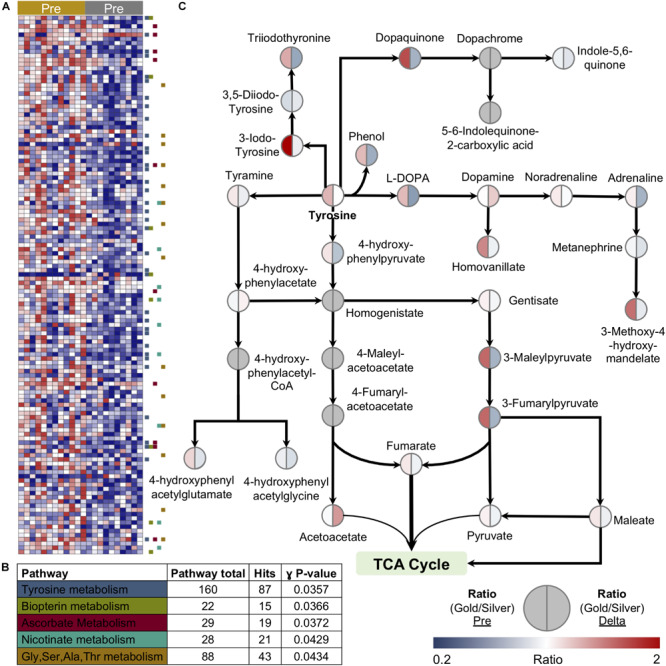
Unbiased metabolomic pathway analysis reveals a link between tyrosine metabolism and lactate levels at performance cutoff. **(A)** A heatmap of features significantly different between the Gold and Silver groups at baseline is shown. **(B)** Pathway analysis using the Recon2 model identified the top five pathways enriched in this discovery dataset. **(C)** Manually validated features within Tyrosine metabolism are shown. The ratio of median values between Gold and Silver groups at baseline is shown on the left side of the circle, while the ratio of the median Delta values (calculated as the Post/Pre fold change) between Gold and Silver groups is shown on the right side of the circle. Values are plotted according to the color scale shown in the lower right.

In an attempt to improve and streamline the analysis of complex biological systems, models have been developed to catalog the system of metabolic reactions (Thiele et al., 2013). Using large unannotated metabolomic datasets, these models hold the potential to efficiently identify networks of metabolic pathways that may be up or downregulated in response to a biological stimulus or setting. In order to gain better insight into basal metabolic differences in cyclists with varying PC levels, we applied an unbiased network analysis using the Recon2 model to the 2,790 putative compounds identified in this study. With a specific focus on compounds that showed distinct levels in the Gold versus Silver groups at baseline (Figure 7A), pathway analysis revealed the significant enrichment of multiple regions of metabolism including tyrosine, biopterin, ascorbate, nicotinate, and glycine/serine/alanine/threonine metabolism (Figure 7B). The most significantly enriched pathway, tyrosine metabolism, is interesting given that it is responsible for catecholamine synthesis, as well as the production of metabolites that can fuel the TCA cycle directly, such as fumarate, or indirectly, such as the ketone body, acetoacetate, and maleate. Subsequent manual interrogation of this pathway highlighted that many of the contained metabolites are elevated prior to exercise in the Gold group, and many are depleted more so in this group than in the Silver group (Figure 7C).

## Discussion

Studies on metabolic adaptations to exercise training in humans have been ongoing for decades. While early studies observed elevated activities and levels of glycolytic and electron transport chain proteins in response to endurance training (Gollnick et al., 1973; Holloszy et al., 1977) more recent work has elucidated the molecular mechanisms of such adaptations [reviewed in Hawley et al. (2018), Hackney (2019)]. Endurance training increases the capacity for lactate consumption in the mitochondria, thus increasing the lactate threshold and athletic output (Brooks, 2018). Lactate clearance capacity is enhanced through increased mitochondrial biogenesis (Little et al., 2010) which is upregulated at the transcriptional level in response to endurance training (Mahoney et al., 2005; Perry et al., 2010) and is controlled in large part by the key mediator, PGC-1α (Kim et al., 2015; Stepto et al., 2012). Increased mitochondrial networks driven by biogenesis, promotion of mitochondrial-rich Type I skeletal muscle fibers (Joyner and Coyle, 2008) and overall upregulations of muscle tissue synthesis, subsequently drive demand for substrates in addition to glucose and lactate including fatty acids and, to a lesser extent, amino acids. Lactate itself may regulate transcriptional activity (San-Millán et al., 2020) possibly through post-translation modifications of histones that alter the epigenomic landscape (Zhang et al., 2019). Future studies in sports and exercise science should focus on the extent of which these latter mechanisms apply to endurance training-mediated physiological adaptations.

The application of metabolomics to understand physiological responses to exercise has led to great advancements in the field of sports physiology over the past decade (Sakaguchi et al., 2019). These investigations have explored the effects of exercise intensity and workout duration on metabolism, as well as metabolic adaptations to chronic training in male and female populations over a range of training status (though female populations have been underrepresented in studies up to the present day). While the vast majority of studies involved amateur subjects with various levels of training, few have included elite or professional athletes assessed either at baseline (single sample) (Al-Khelaifi et al., 2018) or over a 10-day period (Knab et al., 2013). In this study, we analyzed the metabolome of whole blood in 21 international-level World Tour professional male cyclists using samples taken before and after a graded exercise test to exhaustion on a leg cycle ergometer. By normalizing power output to body mass, all cyclists exerted similar effort relative to their body type. Furthermore, the use of identical leg cycle ergometers in an indoor, climate-controlled environment, enabled a controlled monitoring of metabolic responses to exertion in this elite population.

Previously published metabolomics studies have analyzed other biological matrices including serum, plasma, saliva, and urine. While these matrices possess unique advantages in their own right, such as the ability to measure freely circulating metabolites between tissues as is the case with plasma, they disregard the metabolism of circulating blood cells by nature of their collection methods. As appreciation is growing with respect to blood cell adaptations to exercise (Bizjak et al., 2019; de Oliveira Ottone et al., 2019; Estruel-Amades et al., 2019; Shi et al., 2019; Uchida et al., 2019), whole blood analysis enables a more complete representation of physiology. Furthermore, this sample collection is less labor intensive than serum or plasma isolation. The introduction of TAP capillary blood collection devices have enabled field blood collection that does not necessitate traditional venipuncture blood draw (Blicharz et al., 2018) yet does provide comparable quantitative metabolomics data (Catala et al., 2018). Thus, TAP use makes possible rapid sample collection from large subject cohorts, such as participants in cycling races or marathons, thereby fostering metabolomics analyses of active exercise in substantially larger populations than has been previously performed.

Use of whole blood lactate measurements taken during the graded exercise test allowed us to analyze metabolic states of individuals respective to lactate levels at the PC. One advantage of this approach is that it highlighted unique metabolic characteristics between two distinct groups classified based on the amount of lactate produced at a specific power output. This point should be acknowledged when comparing the best performing athletes (Gold group) in this study to the rest of the cohort (Silver group) at the post-exercise time point. However, it is interesting to appreciate that this classification also revealed significant differences at baseline, even prior to the graded exercise test. As the measurements of blood lactate accumulation during a short, graded exercise test can discriminate performance in different groups of cyclists (San-Millán et al., 2009) these results indicate that metabolomic measurements at baseline and during graded exercise testing may serve to expand the predictive qualities of lactate measurement alone. In support, it is noteworthy that the World-Tour cycling season started two weeks after our testing was performed. Many cyclists in the Gold group ended up winning or reaching the podium in the first few races of the season, while riders in the Silver group did not show a great level of performance at the beginning of the year. These results highlight the use of our metabolomics platform as a powerful tool to monitor training status and predict athletic performance.

In agreement with many metabolomics studies, we observed increases in TCA cycle intermediates immediately following exercise. These increases may result from mild ischemia that occurs once oxygen demand exceeds the rate in which it can be supplied by erythrocytes (Zhang et al., 2018). Notably, cyclists with lower PC lactate levels tended to have higher levels of circulating TCA cycle metabolites following exercise. As lactate consumption rate is a marker of mitochondrial content in skeletal muscle (owing in most part to slow-twitch, Type I fibers), TCA cycle metabolite concentrations may also serve as a measure of muscle composition. Surprisingly, cyclists with lower PC lactate had significantly higher levels of the CoA precursor 4’-phospho-pantetheine at baseline, and 4’-phospho-pantothenate after exercise, suggesting upregulation of CoA biosynthesis in these athletes. Notably, these metabolites are intracellular and not readily measurable in plasma. This upregulation may represent a natural adaptation of which previously proposed supplementation strategies have attempted to achieve (Williams, 1989). However, pantothenic acid supplementation alone was shown to have no effect on cycling performance (Webster, 1998) indicating that improved bioavailability, in addition to other adaptations, may be required for the body to upregulate this system.

The preference for carbon sources to fuel the TCA cycle may also depend on lactate threshold. All cyclists exhibited similar mobilization of fatty acids and corresponding acylcarnitines for beta oxidation, of which the medium and long-chain forms tend to be released into circulation predominantly from skeletal muscle during exercise (Makrecka-Kuka et al., 2017). However, cyclists with lower PC lactate in the Gold group demonstrated slightly higher levels of short chain acylcarnitines such as propionylcarnitine and butyrlcarnitine. Propionylcarnitine in particular is released into circulation from the hepato-splanchnic bed after exercise (Xu et al., 2016) pointing to potential liver-mediated adaptations to training that support improved endurance capacity. While short- and odd-chain acylcarnitines can be formed be formed as end products of even- and odd-chain fatty acid oxidation, they are also abundantly produced during the catabolism of certain amino acids, such as branched-chain amino acids (BCAA). Indeed, Gold group cyclists had higher levels of multiple amino acids at baseline, and lower levels of BCAAs leucine, isoleucine, as well as asparagine, after exercise. Amino acids provide small stores of ATP during prolonged exercise (Phillips et al., 1993) and may be dependent on glycogen dynamics (Jackman et al., 1997), which are lactate-dependent (Brooks, 1986). Higher amino acid utilization may also reflect orthogonal mechanisms responsible for protein synthesis (Rundqvist et al., 2013), exercise recovery (Saunders et al., 2007), and overall exercise performance (Kephart et al., 2016). Indeed, skeletal muscle transport of amino acids has been shown to increase in response to training (Roberson et al., 2018).

Amino acid catabolism may serve additional endocrine purposes as well. The upregulation of tyrosine metabolism that is associated with lower PC lactate levels was a particularly interesting finding. Tyrosine is utilized in multiple pathways including protein synthesis, anaplerosis of the TCA cycle, and as a precursor for catecholamine biosynthesis. Catecholamines, including dopamine, norepinephrine, and epinephrine, serve an important role during exercise by mediating sypathoadrenal system function. In addition to cardiovascular and respiratory responses (Zouhal et al., 2008) they modulate hepatic glucose production (Sigal et al., 1996) release of fatty acids from triglycerides (Wahrenberg et al., 1991) and fatigue (Foley and Fleshner, 2008). Indeed, the ratio of dopamine to serotonin decreases with fatigue (Bailey et al., 1993). A study of human metabolic responses to starvation, which offers insight into the physiology of extreme nutrient depletion, observed increased tryptophan consumption (the precursor to serotonin), as well as increased levels of circulating tyrosine, possibly due to decreased utilization (Steinhauser et al., 2018). Meanwhile, pharmacological modulation of serotonin receptors appears to affect time to exhaustion (Meeusen and De Meirleir, 1995). Conversely, aerobic exercise training in animal studies has been show to increase dopamine levels in various brain regions (Foley and Fleshner, 2008). Neuroendocrine control mechanisms of fatigue may have evolved to prevent overexertion (Cordeiro et al., 2017) and as such, these mechanisms should be linked to other mechanisms of endurance capacity in the body. Of note, tyrosine metabolism by monoamine oxidase enzymes is regulated by oxidant stress and glutathione levels (Maker et al., 1981) further suggesting a link between dopamine metabolism, lactate metabolism, and oxidative stress observed in the present study. In this view, it is interesting to note that dopamine (and catecholamines in general) is a direct ROS scavenger (Zhong et al., 2019) and a vasomodulator (Murphy, 2000). As such, dopamine may represent an underappreciated contributor to exercise performance.

In addition to catecholamine biosynthesis, tyrosine could be re-routed for ketogenic roles to provide energy in high endurance trained states. Indeed, levels of tyrosine metabolites were basally higher in the Gold cyclists, and consumed to a larger extent during exertion in conjunction with accumulated levels of acetoacetate. Considering that past reports on tyrosine supplementation have provided mixed results (Chinevere et al., 2002; Tumilty et al., 2011; Watson et al., 2012) future studies using isotopically-labeled tyrosine will help to disentangle its possible catabolic routes with regards to fatigue, lactate clearance capacity, and overall training status.

Glycolysis has long been known as a principal energy generating pathway in tissues due to its high rates of ATP generation under anaerobic conditions. Increased glycolytic markers have also been identified in plasma during exercise (Jacobs et al., 2014). While lactate production as a function of output has been shown to discriminate cyclists of differing training status (San-Millán et al., 2009) all cyclists reached a point of exhaustion just prior to whole blood sampling for metabolomics. As such, only a few glycolytic intermediates trended with rates of lactate accumulation, but none significantly differed between the two groups. Of note, the intra-subject lactate-to-pyruvate ratio is potentially illustrative of performance capacity. Given that this ratio represents an indirect measure of the NAD^+^/NADH ratio, inverse trends between the two groups at baseline and post-test time points is suggestive of specific glycolytic capacity that can drive performance during bouts of high-intensity cycling. Indeed, training strategies centered on high-altitude simulation have shown to be beneficial in promoting glycolysis and work capacity in cyclists (Terrados et al., 1988).

Unlike glycolytic comparisons, the observations of lower PPP intermediates in conjunction with significantly higher levels of oxidant-associated metabolites including oxidized glutathione (GSSG), Met-O, 5-hydroxyisourate and allantoate in the Gold group point to an increased level of basal oxidative stress. Considering that glutathione and PPP intermediates are intracellular metabolites, it is likely that the major contributors to these pools in whole blood are red blood cells (RBCs), by far the most abundant cell in both circulation and the human body (Sender et al., 2016). While exercise promotes erythropoiesis due to increased oxygen demand and need to replenish RBC populations in response to elevated hemolysis (reviewed in Mairbäurl, 2013) effects on RBC circulatory lifespan have only recently been appreciated (Bizjak et al., 2019). One of the hallmarks of RBC aging both in circulation (Lutz and Bogdanova, 2013) and in the blood bank (Nemkov et al., 2015; Yoshida et al., 2019) an environment that accelerates RBC aging (D’Alessandro et al., 2015) is the accumulation of oxidative stress markers including oxidized glutathione and allantoate. Thus, while exercise is known to cause localized inflammation and oxidative stress, systemic markers of such may indeed be indicative of altered hemostasis. In addition to gas exchange, RBC play a multitude of roles in human physiology including regulation of vascular tone, circulatory glucose, lactate, and amino acid content, and catecholamine transport (Nemkov et al., 2018). While it is likely that these characteristics are also important factors in exercise, future work is needed to elucidate the mechanisms RBC use to control physical performance and responses to exercise.

## Conclusion

Here, we report a metabolomics-based investigation of elite, world-competing professional cyclists. Although this group represents a relatively distinct population from the perspective of physical fitness, the robustness and breadth of coverage provided by high-throughput metabolomics highlighted differences with regards to energy and amino acid metabolism, and oxidative stress. These measurements complemented and expanded upon the utility of lactate clearance capacity, which has served as a gold standard to monitor athletic training status. While this study design focused on differences in cyclist oxidative capacity, additional studies can be designed to emphasize the contribution of alternative purely anaerobic energy systems that are needed for very high intensity cycling and sprinting. Future studies using metabolomics-based methodologies should expand steady state measurements by increasing time points, applied to additional forms of exercise that include varying intensity levels and longer endurance periods, and in additional populations with a wider range of physical fitness that are gender- and age-balanced. Despite temporal sampling, steady state measurements lack the molecular resolution necessary to determine metabolic flux. The use of stable isotope tracing with various substrates in the future will allow for a better understanding of the preference for and rates of utilization. Finally, we observed responses in the levels of intracellular metabolites, which in part arise from erythrocytes as these are substantially the most abundant cell population in whole blood. However, while whole blood analysis offers a comprehensive view of physiology, future studies should focus on understanding contribution of different blood components to overall metabolic response to exercise.

## Data Availability Statement

All relevant data is contained within the article.

## Ethics Statement

The studies involving human participants were reviewed and approved by Colorado Multiple Institutional Review Board Protocol ID 17-1281. The patients/participants provided their written informed consent to participate in this study.

## Author Contributions

IS-M, TN, and AD’A designed the experiments. IS-M and TN collected samples. TN, AD’A, and DS acquired and processed the data. TN prepared the figures. TN and IS-M wrote the first draft of the manuscript. All authors commented on the final preparation of the manuscript.

## Conflict of Interest

The authors declare that IS-M, AD’A, and TN are founders of Altis Biosciences LLC and KCH. AD’A and TN are founders of Omix Technologies, Inc. AD’A is a consultant for Hemanext Inc. The remaining authors declare that the research was conducted in the absence of any commercial or financial relationships that could be construed as a potential conflict of interest.

## Supplementary Material

FIGURE S1 Partial Least Squares Discriminant Analysis (PLS-DA) to determine metabolic co-variance between the Gold and Silver cycling groups is shown, along with the metabolites that most strongly contribute to the clustering pattern based on variable importance in projection (VIP) scores.

FIGURE S2 Urea Cycle metabolism. Samples from the Gold (○) and Silver (□) groups are shown Pre and Post exercise test (divided by a dotted line). *p*-values from a two-tailed paired *T*-test of comparisons between the Pre and Post time points using combined Gold/Silver group values are shown above the dashed line. *p*-values from a two-tailed unpaired homoscedastic *T*-test comparing the Gold and Silver groups at each time point are shown on the respective side of the dashed line. **p* < 0.05; ***p* < 0.01; ****p* < 0.001; *****p* < 0.0001.

TABLE S1 A table of the physiological and raw metabolomics data is provided. For the metabolomics data, information is included for the compound name, Compound ID in either KEGG or HMDB, the observed parent mass, median retention time, polarity of detection, and peak area top values in arbitrary units.

TABLE S2 A table of Spearman Correlation Coefficients for the Gold Network, Silver Network, and the Diverging Network (i.e., the difference between the Gold and Silver Networks) is provided.

DATA SHEET S1 A heat map of metabolite hierarchical clustering analysis is shown. Rows and columns were hierarchically clustered according to 1 minus the Spearman rank correlation. Metabolite values are depicted as Z-scores, with the values color coded from row minimum to maximum on a gradient from blue to red, respectively.
