# Supplementary figures and images for: Metabolomics of Endurance Capacity in World Tour Professional Cyclists
Source: Front Physiol. 2020 Jun 5;11:578. doi: 10.3389/fphys.2020.00578 (PMC7291837; doi:10.3389/fphys.2020.00578)

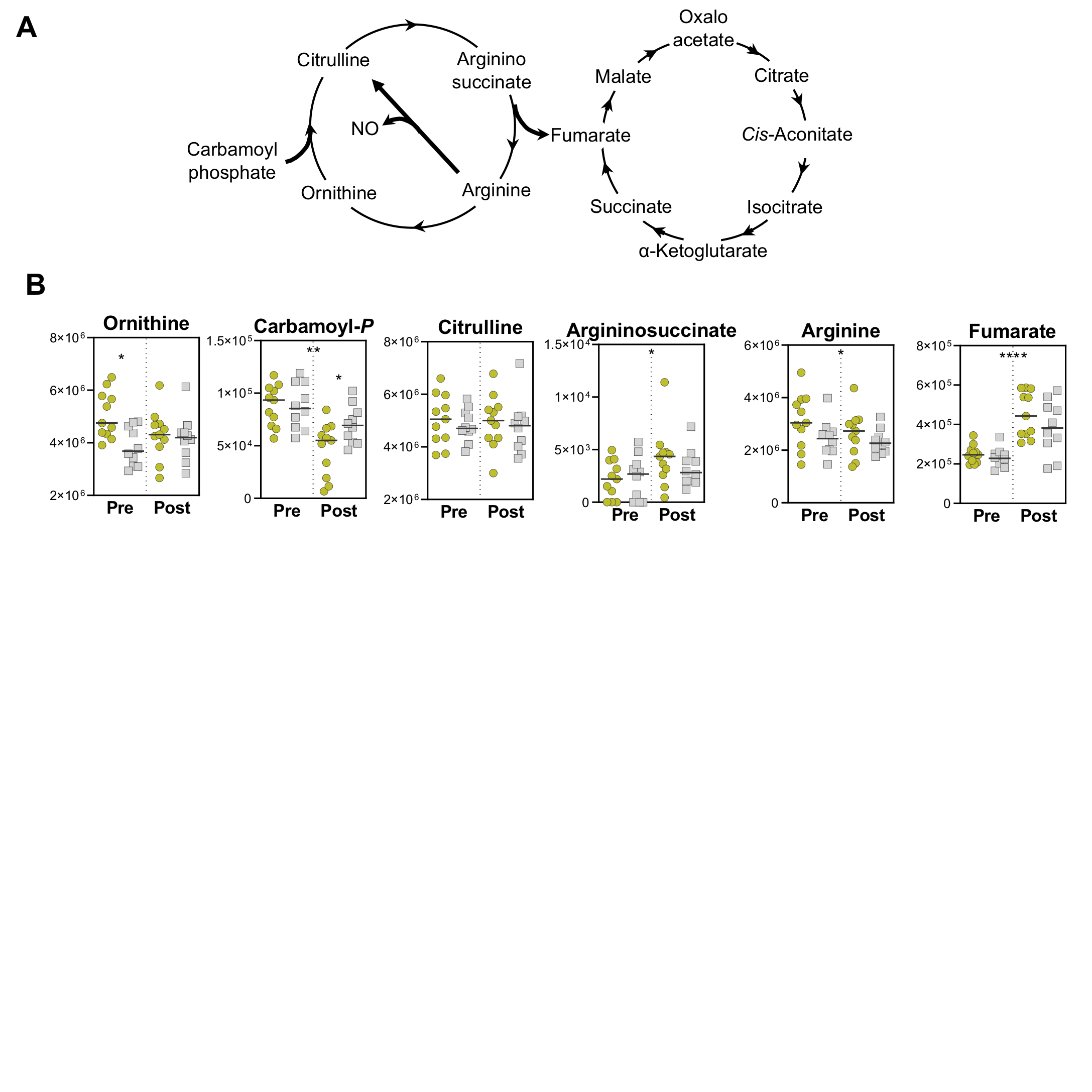

Supplement: FIGURE S1 — Partial Least Squares Discriminant Analysis (PLS-DA) to determine metabolic co-variance between the Gold and Silver cycling groups is shown, along with the metabolites that most strongly contribute to the clustering pattern based on variable importance in projection (VIP) scores. [file Image_1.TIFF]

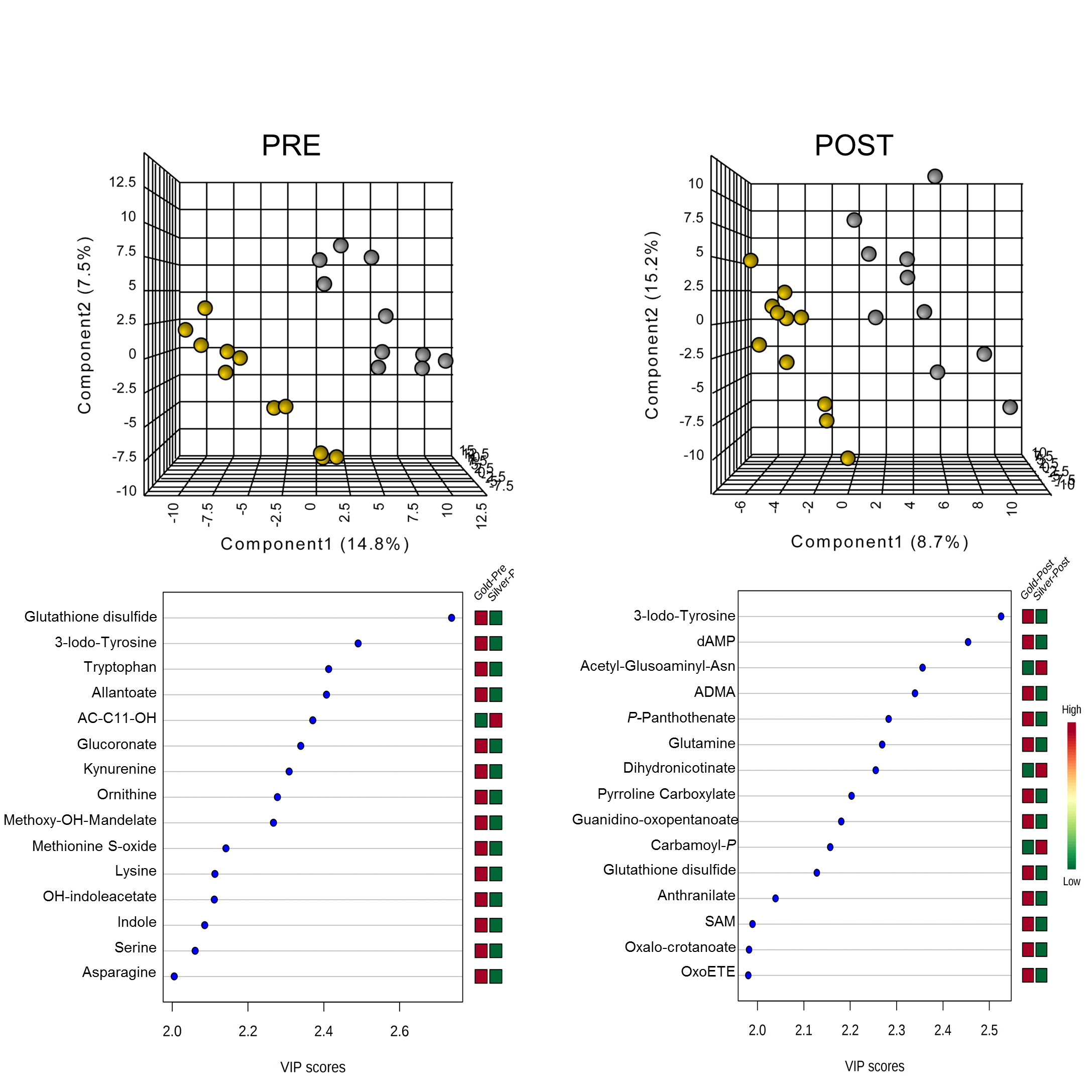

Supplement: FIGURE S2 — Urea Cycle metabolism. Samples from the Gold (○) and Silver (□) groups are shown Pre and Post exercise test (divided by a dotted line). p-values from a two-tailed paired T-test of comparisons between the Pre and Post time points using combined Gold/Silver group values are shown above the dashed line. p-values from a two-tailed unpaired homoscedastic T-test comparing the Gold and Silver groups at each time point are shown on the respective side of the dashed line. *p < 0.05; **p < 0.01; ***p < 0.001; ****p < 0.0001. [file Image_2.TIF]

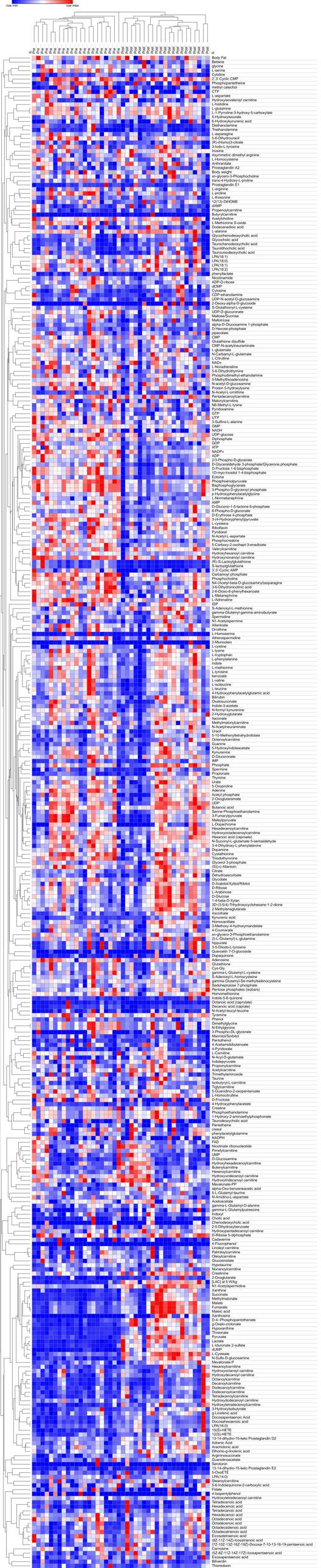

Supplement: DATA SHEET S1 — A heat map of metabolite hierarchical clustering analysis is shown. Rows and columns were hierarchically clustered according to 1 minus the Spearman rank correlation. Metabolite values are depicted as Z-scores, with the values color coded from row minimum to maximum on a gradient from blue to red, respectively. [file Data_Sheet_1.PDF]
